# Silver-cotton nanocomposites: Nano-design of microfibrillar structure causes morphological changes and increased tenacity

**DOI:** 10.1038/srep37320

**Published:** 2016-11-16

**Authors:** Sunghyun Nam, Brian D. Condon, Christopher D. Delhom, Krystal R. Fontenot

**Affiliations:** 1 Southern Regional Research Center, Agricultural Research Service, USDA, 1100 Robert E. Lee Blvd., New Orleans, LA 70124, USA

## Abstract

The interactions of nanoparticles with polymer hosts have important implications for directing the macroscopic properties of composite fibers, yet little is known about such interactions with hierarchically ordered natural polymers due to the difficulty of achieving uniform dispersion of nanoparticles within semi-crystalline natural fiber. In this study we have homogeneously dispersed silver nanoparticles throughout an entire volume of cotton fiber. The resulting electrostatic interaction and distinct supramolecular structure of the cotton fiber provided a favorable environment for the controlled formation of nanoparticles (12 ± 3 nm in diameter). With a high surface-to-volume ratio, the extensive interfacial contacts of the nanoparticles efficiently “glued” the structural elements of microfibrils together, producing a unique inorganic-organic hybrid substructure that reinforced the multilayered architecture of the cotton fiber.

Making nanocomposite fiber involves dispersing secondary-phase nano-sized particles into fiber. Due to the unique properties associated with their size, nanoparticles ranging in composition from silver to various metal oxides have produced functionalized fiber with enhanced or entirely new characteristics^1^,^2^,^3^,^4^,^5^,^6^. The incorporation of nanoparticles is usually achieved by direct mixing with a polymer matrix (or *in*-*situ* polymerization) followed by the spinning process. The agglomeration of nanoparticles therefore is one of the most widely acknowledged challenges facing nanocomposite research^7^. Failure to control the dispersion of nanoparticles cancels out any beneficial effects associated with the nanoscopic dimension and compromises with macroscopic properties.

The incorporation of nanoparticles in natural textile fibers, such as cotton, silk, and wool, has been limited to the surface or near-surface regions of the fiber^8^,^9^,^10^. For improved flame resistance, a trial was run to disperse magnesium hydroxide nanoparticles into cellulose fiber by electrospinning the fiber solution (dissolved in an ionic liquid) mixed with the nanoparticles but suffered significant particle clustering^11^. Such incompatibility of natural polymers with inorganic solids has slowed progress in creating nanoparticle-natural fiber composites and encouraged the development of a new nano-dispersion method. We have previously dispersed silver nanoparticles inside partially dissolved cotton fiber^12^. The technique utilizes the swollen structure of cotton fiber as a nanofluidic system for the controlled synthesis of nanoparticles. The nano-scale reaction volume occupying the opened microfibrillar network provided precise control of reactions, eliminating the need for stabilizing agents to prevent particle agglomeration in the production of silver-cotton nanocomposite fiber.

Fabrication of nanocomposites with desirable properties requires understanding the relationship between nanoparticles and polymer matrices. When nanoparticles are introduced into polymers, they experience enthalpic and entropic interactions, which influence particle size and spatial distribution^7^. To mediate these interactions, various methods, such as surface modification of particles with ligands^13^,^14^, have been applied. Conversely, the addition of nanoparticles has been shown to influence the molecular arrangement of polymer hosts. The resulting modified flow behavior, orientation, or morphology of polymers can be beneficially used in polymer processing or in tailoring the properties of nanocomposites^15^,^16^,^17^. Such cooperative interactions of nanoparticles remains unclear, however, for hierarchically ordered natural polymers. To elucidate how inorganic nanoparticles get along with the organic building blocks of natural fiber, a new integration approach that can serve as an alternative to composite fiber drawing must be devised. Another difficulty involved in establishing the interaction-property relationship for natural fiber composites is the occurrence of intrinsically large variations in the properties of natural fiber, requiring appropriate statistical analysis to determine the influence of nanoparticles.

Here, we showed how silver nanoparticles and the microfibrillar elements of cotton fiber interactively cooperate in nanscale to improve macroscopic performance of the composite fiber. Modifying a previously developed method, we increased the formation of silver nanoparticles within the cotton fiber and created composite assemblies that span multiple layers. The tensile properties of a single nanocomposite fiber were carefully measured, and its failure behavior was well described by the Weibull distribution. Along with the improved mechanical performance, the morphological changes displayed by the nanocomposite fiber suggest that silver nanoparticles, while growing, adhered to the adjacent partially dissolved cellulose chains and improved the structural integrity. Our nanofabrication of the inorganic particle-grown cotton fiber and understanding of its structure-property relationship offer new pathways for nano-designing naturally occurring hierarchy structures to yield a mechanically integrated and functionally durable material in a sustainable manner.

## Results and Discussion

### Nanoparticles grown inside cotton fiber

**Figure 1:**
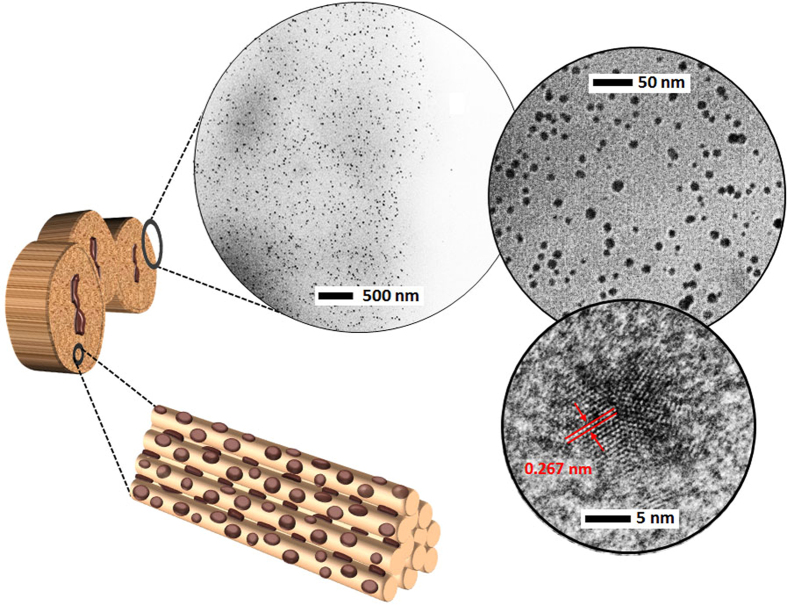
Transmission electron microscopy (TEM) images of the cross-section of silver-cotton nanocomposite fiber showing the internal dispersion of silver nanoparticles and the schematic of the microfibrillar structure of cotton fiber modified by the *in*-*situ* synthesis of the nanoparticles.

**Figure 2:**
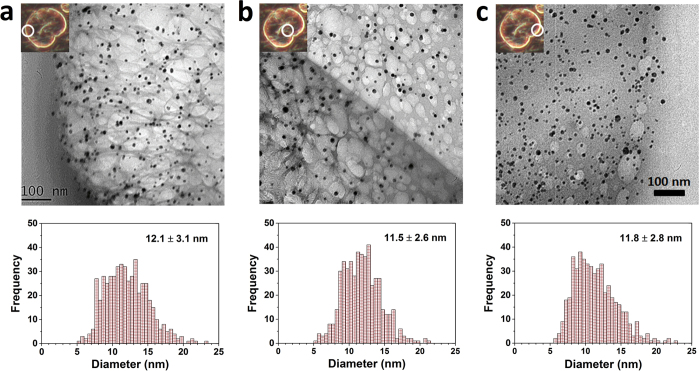
TEM images of the (**a**) left edge, (**b**) center, and (**c**) right edge of the cross-section of silver-cotton nanocomposite fiber taken at the same magnification and corresponding distribution of particle sizes. Particle size was measured by image analysis. The circular morphology appearing in cross-sections was generated by electron-beam damage. Numbers that follow the ± sign are standard deviations.

In the fabrication of silver-cotton nanocomposite fiber, silver ions were diffused into the alkali-swollen cotton fiber to induce their electrostatic binding with the deprotonated cellulose. The obtained self-assembly of the precursors led to the direct formation of nanoparticles on the surface of microfibrils. The reduced flow rate of the reducing agent produced silver nanoparticles inside cotton fiber in a fourfold greater concentration (0.08 wt%). Transmission electron microscopy (TEM) images of the cross-section of the nanocomposite fiber showing the incorporation of nanoparticles and a schematic of the resulting hybrid microfibrillar structure of cotton fiber are shown in Fig. 1. As shown by the TEM images taken from various locations in the cross-section, the nanoparticles were uniformly dispersed throughout the entire volume of fiber; the distributions of particle sizes at the edges and center of the fiber were not significantly different (Fig. 2). The helically oriented microfibrils, whose dimensions are 10–20 nm in width and 20–100 in aspect ratio^18^, created, upon swelling, minute reaction volumes, which are advantageous in controlling the formation of nanoparticles due to the reduced variation in local concentrations of reactants.

**Figure 3:**
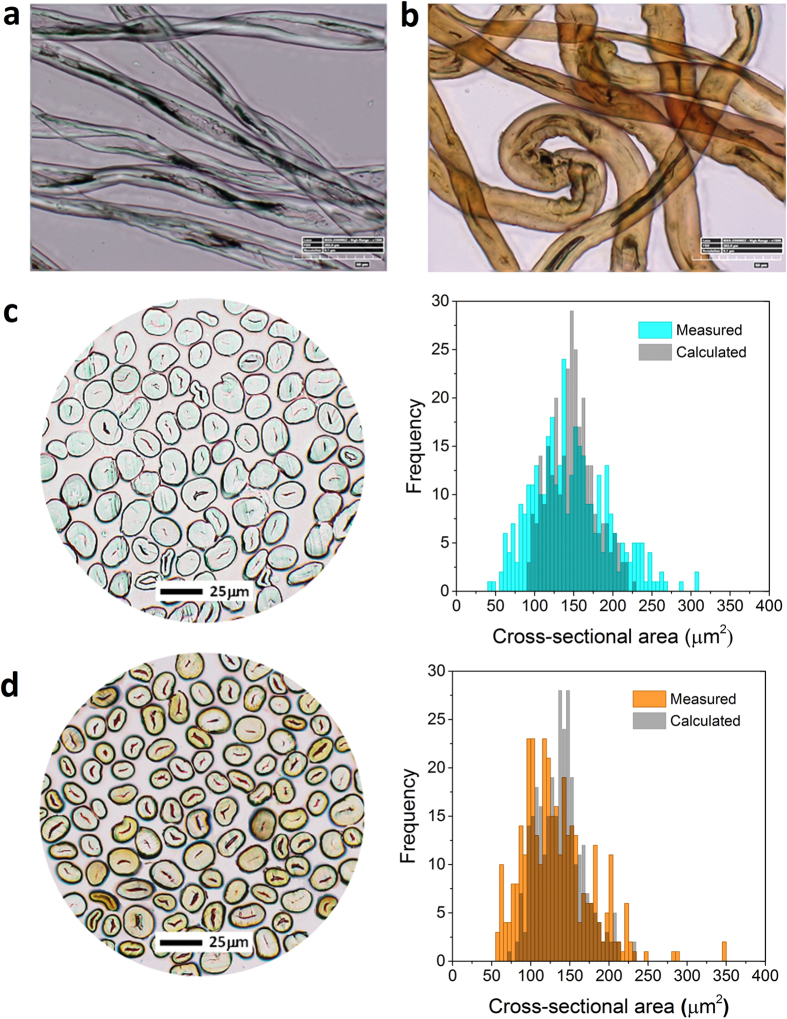
Transmission optical micrographs of the (**a**) control cotton fiber and (**b**) silver-cotton nanocomposite fiber. Optical micrographs of the cross-sections for the (**c**) alkali-treated fiber bundle and (**d**) silver-cotton nanocomposite fiber bundle and corresponding distribution of the cross-sectional areas. The cross-sectional area was measured by image analysis of the micrographs and calculated from the linear density.

One of the intrinsic characteristics of cotton fiber – a ribbon-like shape forming a flat, hollow tube and twists (Fig. 3a) – disappeared for the composite fiber. The alkaline condition used in the particle synthesis swelled the cotton fiber, exhibiting a more circular shape in diameter. The unique optical property of nanoparticles induced by the surface plasmon resonance turned the fiber color from white to brown (Fig. 3b). To investigate the sole effect of nanoparticles, fiber treated with the same condition except for the addition of silver nitrate was included in the characterization (denoted as alkali-treated). When comparing the cross-sections of alkali-treated and nanocomposite fiber bundles (Fig. 3c,d), the cross-sectional shapes were similar to each other but the cross-sectional area of the composite fiber decreased by about 10% (Table 1).

Consistent with the cross-sectional area data, the linear density measured using a vibroscope^19^ was slightly lower for composite fiber (2.15 dtex) than for alkali-treated fiber (2.29 dtex). An alkaline treatment, which shrinks the fiber length by swelling the cell walls, typically increases the linear density of cotton fiber by 17–26%^20^. The distribution of cross-sectional areas calculated from the linear density was narrower and shifted toward smaller values than the distribution of the areas measured by image analysis (Supplementary Fig. S2 and Fig. 3c,d); however, the two measurements agreed in indicating that the formation of nanoparticles suppressed the swelling of fiber. Relatedly, the alkaline treatment decreased the density of the cotton fiber, and the extent of the decrease was reduced with the formation of nanoparticles. The densities measured using helium pycnometry were 1.598, 1.542, and 1.556 g/cm^3^, respectively, for control, alkali-treated, and nanocomposite fibers. Since the weight fraction of silver nanoparticles was very small, no significant contribution of the nanoparticles to the density of the composite was assumed. In fact, the density of the composite (1.543 g/cm^3^) calculated by a binary mixing equation (Supplementary Eq. S1) was similar to the density of the alkali-treated fiber. Therefore, the deviation of the measured density from the calculated density implies differences in structural organization of the cotton fiber.

### Enhanced structural integrity

These results raise the question whether the reduced swelling in the nanocomposite fiber is associated with a lower degree of alkalization (mercerization). According to the mercerization mechanism^21^, at a high enough concentration an alkali solution can enter the crystalline region of cellulose fiber to cause a crystal-to-crystal phase transformation: the native cellulose Iβ first transforms into Na-cellulose Iβ and then to the more stable Na-cellulose II. However, due to its lower energy, the cellulose II remains after washing sodium ions away.

**Figure 4:**
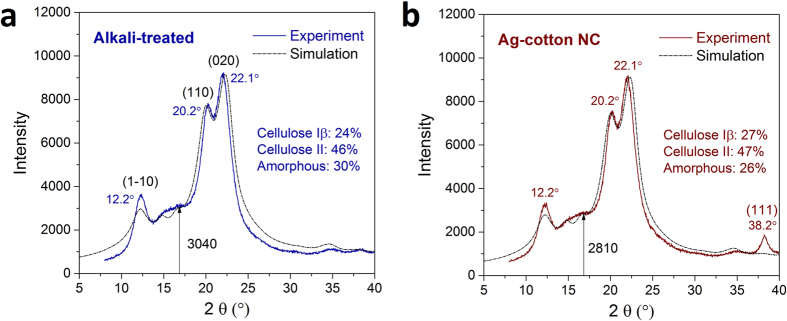
X-ray diffraction patterns of (**a**) alkali-treated cotton fiber and (**b**) Ag-cotton NC fiber plotted with the corresponding simulated pattern. The respective percentages were determined based on the ternary mixing of the calculated patterns of cellulose Iβ, cellulose II, and amorphous cellulose (Fig. S1). Experimental details are provided in the Supplementary Information.

In the *in*-*situ* synthesis of silver nanoparticles, silver complex ions were introduced at the Na-cellulose II stage; therefore, it was assumed that the polymorphic conversion to cellulose II would be intact. For confirmation, X-ray diffraction patterns for alkali-treated and nanocomposite fibers are compared in Fig. 4. In addition to the main peaks of the cellulose polymorphs, a small, broad peak at 38.2° 2θ from the (111) plane of silver crystal was observable. The (110) peak, which was located at 16.5° 2θ for cellulose Iβ (control cotton) (Figs S1 and S3), shifted to 20.1° 2θ, indicating the conversion from cellulose Iβ to cellulose II. But the relatively low intensity of the (110) peak indicates incomplete conversion. The degree of conversion was measured by a previously developed simulation method^22^ that is based on ternary mixing of the calculated diffraction patterns of cellulose Iβ, cellulose II, and amorphous cellulose of cotton fiber (Eqs S3 and 4). The percentages of cellulose II for alkali-treated and nanocomposite fibers, 46% and 47%, respectively, were almost the same, showing an insignificant effect of the particle synthesis on polymorphic conversion. However, as reflected by the decreased intensity between the (1–10) and (110) peaks (arrows in Fig. 4a,b), the calculated amorphous fraction of the composite fiber (26%) was smaller than that of the alkali-treated fiber (30%). It should be noted that these amorphous fractions were much higher than 10% for the control cotton fiber (Fig. S3). These results suggest that, upon removal of sodium ions, the inter- and intra-molecular hydrogen bonds cleaved by swelling could not be fully recovered, resulting in the loosening of the original structure, compromising its integrity (supported by the previously reported density data). For the nanocomposite fiber, the ion exchange with silver ions influenced the recrystallization in a positive way.

### Nanoparticle-reinforced cotton fiber

The influence of the modified internal structure on the macroscopic properties of nanocomposite fiber was examined by directly measuring its tensile properties at the individual fiber level - a few millimeters in length and tens of microns in width. The tenacity (*σ*) (i.e., the breaking force divided by the linear density) of the nanocomposite fiber was higher than that of control and alkali-treated cotton fibers, indicating the reinforcing effect of nanoparticles (Table 1). This reinforcement was more evident with regard to breaking strength, which was calculated using cross-sectional areas.

**Figure 5:**
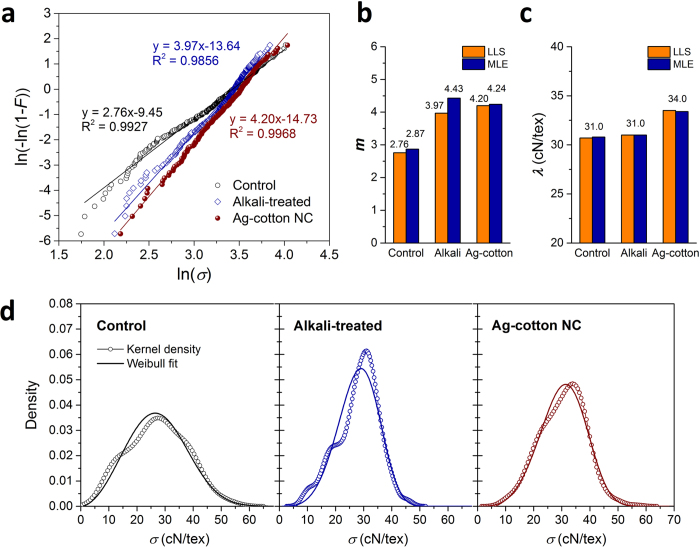
(**a**) Weibull plots of tenacity for control cotton fiber, alkali-treated cotton fiber, and Ag-cotton NC fiber using the linear least squares (LLS) method. Straight lines represent the linear fit of Equation (S10). Comparisons of (**b**) Weibull modulus (*m*) and (**c**) scale parameter (*λ*) values determined by the LLS and maximum likelihood estimation (MLE) methods. (**d**) Nonparametric kernel density of tenacity plotted with the Weibull density, whose parameters were determined by the MLE.

The scattering behavior of tenacity was analyzed using the two-parameter Weibull model (Eq. S5)^23^. The Weibull distribution, which is based on the weakest-link theory^24^, has usefully explained the strength variation of natural lignocellulosic fibers^25^. The two parameters – the scale parameter (*λ*) and the Weibull modulus (shape parameter) (*m*) – were determined using the linear least squares (LLS) and maximum likelihood estimation (MLE) methods. As obtained using the LLS method (Eq. S10), the linear fits of the Weibull model with the control, alkali-treated, and nanocomposite fibers are presented in Fig. 5a. The nanocomposite fiber exhibited excellent fit over the entire range of tenacity, whereas control and alkali-treated fibers showed some deviation at low tenacities. The distinct slopes and intercepts observed in the linear fits, which determine *m* and *λ*, respectively, imply differences in failure behavior. The *m* was ordered in the following sequence: nanocomposite > alkali-treated > control (Fig. 5b), indicating that the formation of nanoparticles under the alkaline condition reduced the dispersion of the defects within the fiber.

The *m* determined by the MLE method (Eqs S11–17) was slightly higher than the value obtained by the LLS method. The MLE method agreed with the LLS method regarding the enhanced consistency in strength by alkalization, but did not agree regarding the influence of nanoparticles, i.e., the decreased *m* for the nanocomposite fiber. Considering the high precision of MLE in the estimation of the Weibull modulus^26^, the MLE method was suggested for comparison of *m* in alkali-treated and nanocomposite fibers. More convincingly, the relationships of *m* as derived from the MLE with tenacity and elongation – the higher the *m* the lower the tenacity and the lower the elongation (Table 1) – are consistent with the results reported in the literature^27^. On the other hand, the *λ* values determined by the two methods were consistent (Fig. 5c). The mean tenacities calculated by *λ* (Eq. S8) showed excellent agreement with the experimental averages for all fibers (Table 1).

The relatively more stretchable alkali-treated fiber can yield plastic deformation against tensile stress, which causes some inconsistency with the weakest-link concept for the Weibull distribution. As a result, the alkali-treated fiber exhibited a greater discrepancy between the nonparametric kernel density of the tenacity and the Weibull estimation (Fig. 5d). For quantitative evaluation, the maximum deviation (*D*_*KS*_) of the Weibull distribution from the empirical distribution (Eqs S6 and 7) was obtained by the Kolmogorov-Smirnov test (Eq. S18). The *D*_*KS*_ value for alkali-treated fiber was 0.0602, which was higher than those for the control and nanocomposite fibers, which were 0.0416 and 0.0398, respectively. However, it should be noted that the *D*_*KS*_ values for all these fibers were lower than the respective critical values (Table 1), signifying the goodness of the Weibull fits, which was also apparent in the cumulative distribution plots (Fig. S5). The smallest *D*_*KS*_ value observed for the nanocomposite fiber suggests that its strength became more dependent on the distribution of flaw sizes – a characteristic of brittle failure. This change in failure behavior was attributed to the restricted micro- and macro-fibrillar mobilities caused by the fixation of nanoparticles.

### Morphological evolution

**Figure 6:**
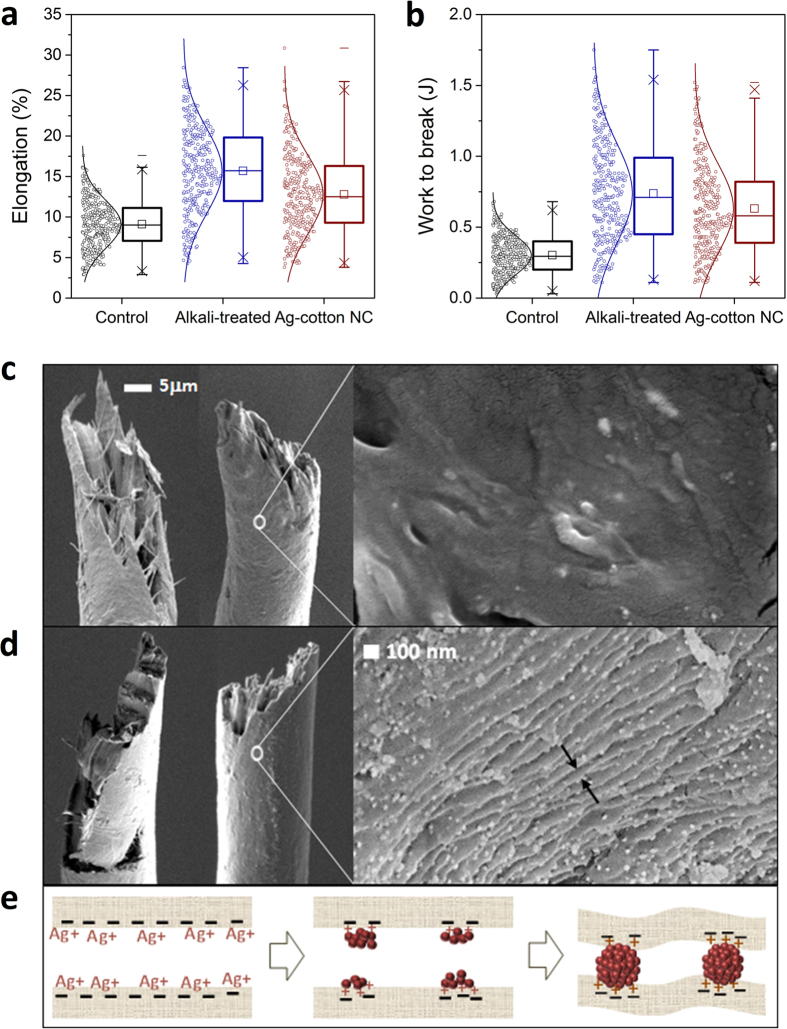
Box plots and distributions of (**a**) elongation at break and (**b**) work to break with Gaussian fit for control cotton fiber, alkali-treated cotton fiber, and Ag-cotton NC fiber. Scanning electron microscopy (SEM) images of the fracture morphology after textile tests for (**c**) alkali-treated cotton fiber and (**d**) Ag-cotton NC fiber taken at the same magnification. A close-up of the fiber surface was obtained by field emission SEM. (**e**) Schematic drawing illustrating how the *in*-*situ* synthesis of silver nanoparticles within the partially dissolved cotton evolved the nanoscopic surface ripples.

The restricted motion of the structural elements of the nanocomposite fiber was supported by the measurements of the elongation at break and work to break (Fig. 6a,b). The alkali-treated fiber, which had shrunk along the fiber length, exhibited a 72% increase in elongation at break as compared with the control fiber. The increased amorphous fraction and loosened structure as a result of the alkaline treatment introduced ductile characteristics, which resulted in requiring 2.5 times more energy to break the fiber. The distributions of both elongation at break and work to break substantially broadened after the alkaline-treatment. The formation of nanoparticles in the alkali-swollen fiber reduced the elongation by 18%. The insertion of nanoparticles between the microfibrils created a rigid interface that reduced structural deformability. Consequently, the work to break for the nanocomposite fiber decreased slightly. The unique fabrication method applied to nanocomposite fiber, that is, opening the hierarchical structure followed by the reorganization, transformed the cotton fiber, giving it unexpected tensile properties - greater strength and higher elastic strain.

The modified internal structure of the nanocomposite fiber was manifest in the fracture morphology. Optical micrographs of the fractured alkali-treated fiber following tensile tests showed the frayed separation of microfibrils along the fiber axis (Fig. 6c). This irregular axial splitting was caused by the unwinding of the region, where the helix angle of the microfibrillar orientation was changed, relieving the tensile stress^28^. A similar morphology of breaks was also observed for the control fiber (Fig. S4). The nanocomposite fiber however exhibited different fracture behavior, i.e., a clean fracture across the fiber diameter (Fig. 6d), indicating that the nanoparticles hindered the untwisting of the helical orientation. The resulting resistance to elongation was supported by the decreased percentage elongation at break. With a high surface-area-to-volume ratio, nanoparticles formed extensive interfacial contacts with microfibrils and induced efficient load transfer between them. A close-up of the surface of the nanocomposite fiber shown in Fig. 6d (right) shows nanoscopic surface ripples and the uniform dispersion of silver nanoparticles. While growing, nanoparticles interacted with the partially dissolved cellulose chains to produce a unique hybrid substructure, where the nanoparticles “glued” the neighboring microfibrills (Fig. 6e). The formation of a new substructure therefore reduced the swelling along the radius of the fiber and generated the wrinkled surface.

## Conclusion

We have reported for the first time how inorganic nanoparticles interact with a hierarchically ordered natural polymer. Through *in*-*situ* synthesis within partially dissolved cotton fiber, extremely small silver nanoparticles (12 ± 3 nm in diameter) were grown between the helically oriented microfibrils. While growing, the nanoparticles electrostatically bound the microfibrils to improve the structural organization and change the fiber morphology. The reinforcement provided by the nanoparticles was demonstrated by increased tenacity, and the brittle failure behavior of the nanocomposite fiber was well described by the Weibull distribution. Notably, the concentration of nanoparticles exhibiting such influence on the macroscopic properties was less than 0.1% based on fiber weight. The large surface area of the nanoparticles resulting from the uniform dispersion is responsible for the maximized functionality observed. Such newly modified cotton fiber, in which functional nanoparticles were not only chemically attached but also physically trapped in the microfibrillar network, can be fabricated into nanoengineered textiles with permanent functions or designed into advanced devices that release functionalities under appropriate conditions in a controlled manner over a prolonged period of time.

## Methods

### Preparation of Ag-cotton NC fiber

Sodium hydroxide (NaOH, 97%), silver nitrate (AgNO_3_, 99.9%), ammonium hydroxide solution (NH_4_OH, 28–30%), and L-ascorbic acid (C_6_H_8_O_6_, 99%) were purchased from Sigma Aldrich. All chemicals were used as received without further purification. American upland raw cotton was acquired from the national registry and mechanically cleaned without using water and chemicals through re-ginning and lint-cleaning processes. 0.5 g of cotton fiber, which had been scoured, was immersed in 15 ml of 23% sodium hydroxide solution at room temperature for 15 min. The NaOH solution was then extracted from the fiber by centrifugation to achieve 230% wet-pick-up. The alkali-swollen fiber was transferred to 15 ml of 0.03 M silver nitrate aqueous solution, and while the solution was being agitated, ammonium hydroxide solution was added drop-wise until the solution became clear. The reduction reaction was then carried out by the addition of 0.01 M L-ascorbic acid with a flow rate of 0.05 ml/min for 30 min. The solution, which was subjected to vigorous agitation, turned yellow immediately and then black. The obtained Ag-cotton NC fiber was washed using a detergent (0.15 wt%) for 24 hours, rinsed multiple times for 48 hours, and air-dried.

### Characterization

The concentration of silver nanoparticles in the nanocomposite fiber was measured by neutron activation analysis (Elemental Analysis, Inc., Lexington, KY). The formation of silver nanoparticles within the fiber was examined using a TEM (JEOL 2010) operating at 200 kV. For the sample preparation, a bundle of combed fibers was embedded in a mixture of methyl methacrylate and butyl methacrylate, which was polymerized using a UV cross-linker (UVP, CL-1000) for 30 min following the published technique^29^,^30^. A block of the fiber bundle was cut into approximately 100 nm-thick slices using a PowerTome Ultramicrotome (Boeckeler Instruments, Inc). The obtained sections were placed on a carbon-film-coated copper grid, and the embedding medium was removed with methyl ethyl ketone. The optical microscopic images of samples were taken in the transmission mode using a digital microscope (KH-8700, Hirox). Nanoparticle size and cross-sectional areas of fiber were measured using Image J software.

The density of the fiber was measured by helium pycnometery (AccuPyc II 1340, Micromeritics)^31^ at 20 °C. About 0.11 g of fiber bundle was placed in a sample cell, the volume of which was 1.33 cm^3^. The pressure difference in the helium gas in the reference cell, after the gas was released to the sample cell, was used to determine the volume of the fiber and thus its density.

X-ray diffraction (XRD) measurement was conducted using an XDS 2000 diffractometer (Scintag Inc.). Approximately 0.15 g of the ground sample was pressed with 127 MPa of pressure in a hydraulic press and cut into a circular disc 2.5 cm in diameter. The diffraction pattern of the sample was recorded using Cu-Ka radiation generated with 43 kV and 38 mA (1.54056 Å) at a scan rate of 0.6°/min. The degree of conversion to cellulose II was determined by the simulation of XRD patterns with the calculated patterns of cellulose Iβ, cellulose II, and amorphous cellulose for cotton fiber (Fig. S1). Using the coordinates of the asymmetric units of cellulose I and cellulose II, the diffraction patterns of their completely periodic crystals were constructed with the Mercury 3.0 program. The full width at half maximum (FWHM) values - 1.54°, 2.3°, and 9° - were set for cellulose Iβ, cellulose II, and amorphous cellulose, respectively, which were experimentally obtained from control cotton, fully mercerized cotton, and ball-milled cotton. A binary (Eq. S2) and ternary mixing equations (Eqs S3 and 4) were used for control cotton and alkali-treated cotton samples, respectively.

The linear density (*L*) and tensile properties of the fiber were measured using a Favimat tester (Textechno H. Stein GmbH). A single fiber was clamped with a gauge length (*l*) of 0.32 cm and pre-tensioned with force (*T*) of 0.85 cN. According to the vibroscopic technique (ASTM D 1577), the *L* (g/cm = 10^−6^ dtex) was determined by the resonant frequency of transverse vibration of the fiber using the following equation:


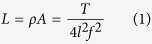


where *ρ*, *A*, and *f* are the density of fiber (g/cm^3^), the cross-sectional area of fiber (cm^2^), and the resonant frequency (Hz), respectively. The same fiber section was extended at a cross-head speed of 3.2 mm/min to measure breaking force, percentage elongation at break, and work to break. The tensile force precision was within a range of 1 mN.

## Additional Information

**How to cite this article**: Nam, S. *et al.* Silver-cotton nanocomposites: Nano-design of microfibrillar structure causes morphological changes and increased tenacity. *Sci. Rep.* **6**, 37320; doi: 10.1038/srep37320 (2016).

**Publisher’s note**: Springer Nature remains neutral with regard to jurisdictional claims in published maps and institutional affiliations.

## Supplementary Material

Supplementary Information

## Figures and Tables

**Table 1:** Comparison of macroscopic properties of control cotton fiber, alkali-treated cotton fiber, and silver-cotton nanocomposite fiber (noted as Ag-cotton NC).

	Control	Alkali-treated	Ag-cotton NC
No. of fibers tested	308	303	306
Linear density (dtex)	1.99 ± 0.35a^1^	2.29 ± 0.44b	2.15 ± 0.45c
Density (g/cm^3^)	1.598 (1.554)^2^	1.542 (1.530)^2^	1.556 (1.543)^3^
Measured cross-sectional area (μm^2^)	120.8 ± 36.0a	146.2 ± 48.9b	132.1 ± 44.6c
Calculated cross-sectional area (μm^2^)	123.9 ± 20.4a	148.4 ± 28.7b	137.9 ± 29.0c
Tenacity (cN/tex)	27.3 ± 10.5a	28.0 ± 7.4a	30.4 ± 8.1b
Breaking strength^4^ (MPa)	437.6 ± 167.9a	432.4 ± 114.3a	473.2 ± 125.3b
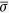 ^5^ (cN/tex)	27.4 (37.5)^6^	28.3 (22.8)	30.4 (28.6)
*D*_*KS*_	0.0416 (0.0775)^7^ (0.6611)^8^	0.0602 (0.0781) (0.2222)	0.0398 (0.0777) (0.7173)
Breaking elongation (%)	9.11 ± 2.96a	15.68 ± 5.22b	12.78 ± 4.86c
Work to break (J)	0.30 ± 0.14a	0.74 ± 0.35b	0.63 ± 0.31c

^1^Averages followed by different letters are significantly different (p < 0.001) based on Tukey’s multiple comparison test; ^2^Value taken from the literature^32^; ^3^Value calculated by a binary mixing rule; ^4^Calculated using cross-sectional areas obtained from linear density; ^5^Calculated from Weibull parameters (Eq. S8); ^6^Coefficient of variation calculated from Weibull modulus (Eq. S9); ^7^Kolmogorov-Smirnov critical value with a significance level of 0.05; ^8^p-value; A number that follows the ± sign is a standard deviation.
