# Supplementary material for: Silver-cotton nanocomposites: Nano-design of microfibrillar structure causes morphological changes and increased tenacity
Source: Sci Rep. 2016 Nov 16;6:37320. doi: 10.1038/srep37320 (PMC5110977; doi:10.1038/srep37320)
Supplement: Supplementary Information [file srep37320-s1.pdf]

Supporting Information

Silver-cotton nanocomposites: Nano-design of microfibrillar structure causes morphological changes and increased tenacity

Sunghyun Nam, Brian D. Condon, Christopher D. Delhom, and Krystal R. Fontenot

Southern Regional Research Center, Agricultural Research Service, USDA, 1100 Robert E. Lee Blvd., New Orleans, LA 70124, USA.

Correspondence should be addressed to Dr. Sunghyun Nam, email: sunghyun.nam@ars.usda.gov

Supplementary Methods

Scouring. Scouring was carried out by agitating cotton fiber in an aqueous solution containing NaOH (1.8 g/L) and Triton X-100 (0.2 g/L) with a liquid-to-fiber ratio of 22.4:1 at 100 °C for 60 min. After the treatment, the fiber was washed in circulating water at 100 °C for 20 min, followed by cold water for 20 min. The scoured fiber was then neutralized with a solution of acetic acid (0.25 g/L) for 10 min, rinsed multiple times, and air-dried.

Density. The density of the nanocomposite fiber was calculated based on the weight fractions of fiber and nanoparticles using a binary mixing equation:

$$\frac{1}{\rho_{NC}} = \frac{w_f}{\rho_f} + \frac{w_{NP}}{\rho_{NP}} \quad \text{(S1)}$$

where ρ_{NC}, ρ_f, ρ_{NP} are the densities of the nanocomposite fiber, the fiber matrix, and the nanoparticles, respectively, and w_f and w_{NP} are the weight fractions of the fiber and nanoparticles, respectively.

Percentages of cellulose Iβ, cellulose II, and amorphous cellulose. The crystallinity and the extent of conversion to cellulose II were determined by the simulation of XRD patterns with the calculated patterns of cellulose Iβ, cellulose II, and amorphous cellulose for cotton fiber. The diffraction pattern of control cotton was calculated by the following equation:

$$I = w_c I_c + (1 - w_c) I_a \quad \text{(S2)}$$

where w_c is the fraction of crystalline cellulose and I_c and I_a are the intensities of crystalline and amorphous celluloses, respectively. For partially mercerized cotton (i.e., alkali-treated cotton and Ag-cotton NC fiber), a ternary mixing equation was used:

$$I = w_{c,I\beta} I_{c,I\beta} + w_{c,II} I_{c,II} + w_a I_a \quad \text{(S3)}$$

$$w_a = 1 - w_{c,I\beta} - w_{c,II} \quad \text{(S4)}$$

where $w_{c,I\beta}$, $w_{c,II}$, and w_a are the fractions of the cellulose Iβ, cellulose II, and amorphous cellulose, respectively, and $I_{c,I\beta}$, $I_{c,II}$, and I_a are the intensities of the crystalline cellulose Iβ, crystalline cellulose II, and amorphous cellulose, respectively.

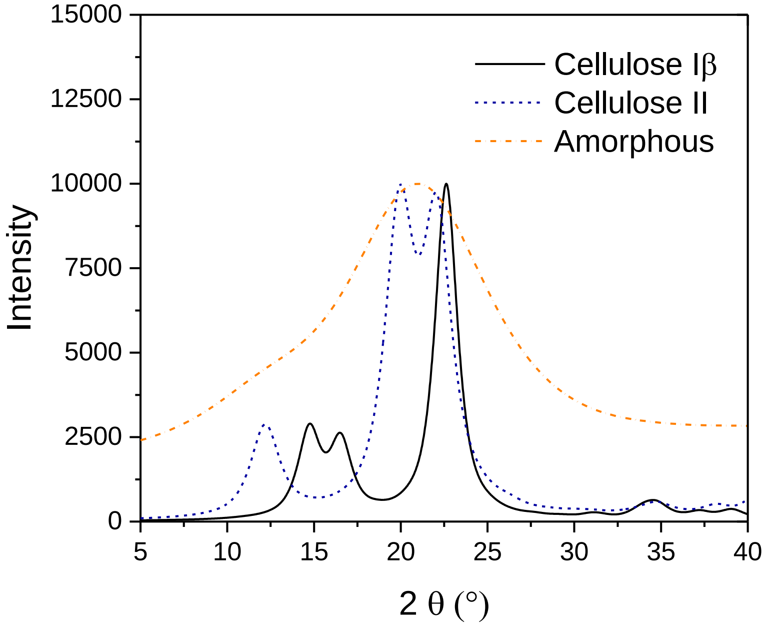

Figure S1. Calculated diffraction patterns of cellulose Iβ, cellulose II, and amorphous cellulose of cotton fiber.

Fiber morphology. To examine the fracture morphology, an additional tensile test was performed using a stelometer. Prior to the test, the cotton samples were equilibrated for 48 hours at 70±2 °F and 65±2% RH. Approximately 0.1 mg of fiber was hand-combed and passed through a fine comb. The resulting sample was secured in a Pressley clamp with a 1/8 inch gauge and tested under standard conditions following the ASTM D-1445-75 method (the standard test method for breaking strength and elongation of cotton fibers – the flat bundle method). The fractured samples were coated with a gold palladium alloy and observed using a scanning electron microscope (SEM) (Philips, XL 30) operating at 5-7 keV and a beam current of 0.5 nA. The surface morphology was observed at high magnifications using field emission SEM (LEO 1530 VP).

Weibull distribution. The two-parameter Weibull cumulative distribution (F) for tenacity (σ) is:

$$F(\sigma;\lambda,m)=1-\exp\left[-\left(\frac{\sigma}{\lambda}\right)^{m}\right] \tag{S5}$$

where λ is the scale parameter and m is the shape parameter (Weibull modulus). These two unknowns can be estimated from the empirical distribution function (F_n) for n independent and identically distributed tenacity values.

$$F_n(\sigma)=\frac{1}{n}\sum_{i=1}^{n} I_{\{\sigma_i\le\sigma\}} \tag{S6}$$

where I is the indicator function, equal to 1 for $\sigma_i \le \sigma$ and 0 otherwise. This empirical distribution can be commonly obtained by the following equation:

$$F_n(\sigma)=\frac{i}{n+1} \tag{S7}$$

where n is the total sample size, and i is the index of the tenacity value when the tenacity data are arranged in ascending order. From the determined parameters, the theoretical average ($\bar{\sigma}$) and coefficient variation of tenacity (CV) can be calculated by the following equations, respectively:

$$\bar{\sigma}=\lambda\Gamma\left(1+\frac{1}{m}\right) \tag{S8}$$

$$CV=\frac{\left[\Gamma\left(1+\left(2/m\right)\right)-\Gamma^{2}\left(1+\left(1/m\right)\right)\right]^{1/2}}{\Gamma\left(1+\left(1/m\right)\right)} \tag{S9}$$

where $\Gamma(x)=\int_{0}^{\infty} t^{x-1}e^{-t}dt$ is the gamma function.

For the LLS method, the following linear form is obtained by taking double natural logarithms of both sides of equation (S5):

$$\ln\left[\ln\left(\frac{1}{1-F_e(\sigma)}\right)\right]=m\ln\sigma-m\ln\lambda \tag{S10}$$

where the m is directly obtained from the slope, and the λ is deduced from the intercept in the liner fit.

For the MLE method, the likelihood function (L) of n random measurements is obtained as:

$$L(\sigma_1,...,\sigma_n;m,\lambda)=\prod_{i=1}^{n}\left[\left(\frac{m}{\lambda}\right)\left(\frac{\sigma_i}{\lambda}\right)^{m-1}\exp\left(-\left(\frac{\sigma_i}{\lambda}\right)^{m}\right)\right] \tag{S11}$$

$$L(\sigma_1,...,\sigma_n;m,\lambda)=\left(\frac{m}{\lambda^m}\right)^{n}\exp\left[-\frac{1}{\lambda^m}\sum_{i=1}^{n}\sigma_i^m\right]\prod_{i=1}^{n}\sigma_i^{m-1} \tag{S12}$$

The λ and m can be determined when the value of the measurement is most likely to occur, that is, the L is maximized. This optimization can be obtained by using the log-likelihood function for easier computation.

$$\ln L\left(\sigma_1,\ldots,\sigma_n;m,\lambda\right)=n\ln\left(m\right)-nm\ln\left(\lambda\right)-\frac{1}{\lambda^m}\sum_{i=1}^{n}\sigma_i^m+\left(m-1\right)\sum_{i=1}^{n}\ln\left(\sigma_i\right) \tag{S13}$$

Subsequently, the partial derivatives of ln L with respect to m and λ are set to zero.

$$\frac{\partial\ln L}{\partial m}=\frac{n}{m}-n\ln\left(\lambda\right)+\frac{\ln\lambda}{\lambda^m}\sum_{i=1}^{n}\sigma_i^m-\frac{1}{\lambda^m}\sum_{i=1}^{n}\sigma_i^m\ln\left(\sigma_i\right)+\sum_{i=1}^{n}\ln\left(\sigma_i\right)=0 \tag{S14}$$

$$\frac{\partial\ln L}{\partial\lambda}=-\frac{nm}{\lambda}+\frac{m}{\lambda^{m+1}}\sum_{i=1}^{n}\sigma_i^m=0 \tag{S15}$$

Equations (S14) and (S15) yield simplified likelihood equations, respectively, for m and λ:

$$m=\left[\frac{\sum_{i=1}^{n}\sigma_i^m\ln\left(\sigma_i\right)}{\sum_{i=1}^{n}\sigma_i^m}-\frac{1}{n}\sum_{i=1}^{n}\ln\left(\sigma_i\right)\right]^{-1} \tag{S16}$$

$$\lambda=\left[\frac{1}{n}\sum_{i=1}^{n}\sigma_i^m\right]^{\frac{1}{m}} \tag{S17}$$

which can be numerically solved.

Goodness-of-fit test. The Kolmogorov-Smirnov goodness-of-fit test was performed to examine whether the obtained tenacity data can be described by the Weibull distribution. Under the hypothesis that the data follow the Weibull distribution, the test statistic (D_{KS}), which is the greatest vertical distance between the empirical and Weibull distributions, can be measured:

$$D_{KS} = \sup_{\sigma} \left| F_n(\sigma) - F_{m,\lambda}(\sigma) \right| \tag{S18}$$

where $F_n(\sigma)$ is the empirical distribution and $F_{m,\lambda}(\sigma)$ is the Weibull distribution with the parameters determined from the MLE method. When the hypothesis is true for a large sample size, D_{KS} is distributed with its own distribution function. Therefore, if the hypothesis is true, D_{KS} is smaller than the critical value (D_C) determined from a significance level (α). $\alpha = 0.05$ was used in this study.

Supplementary Results

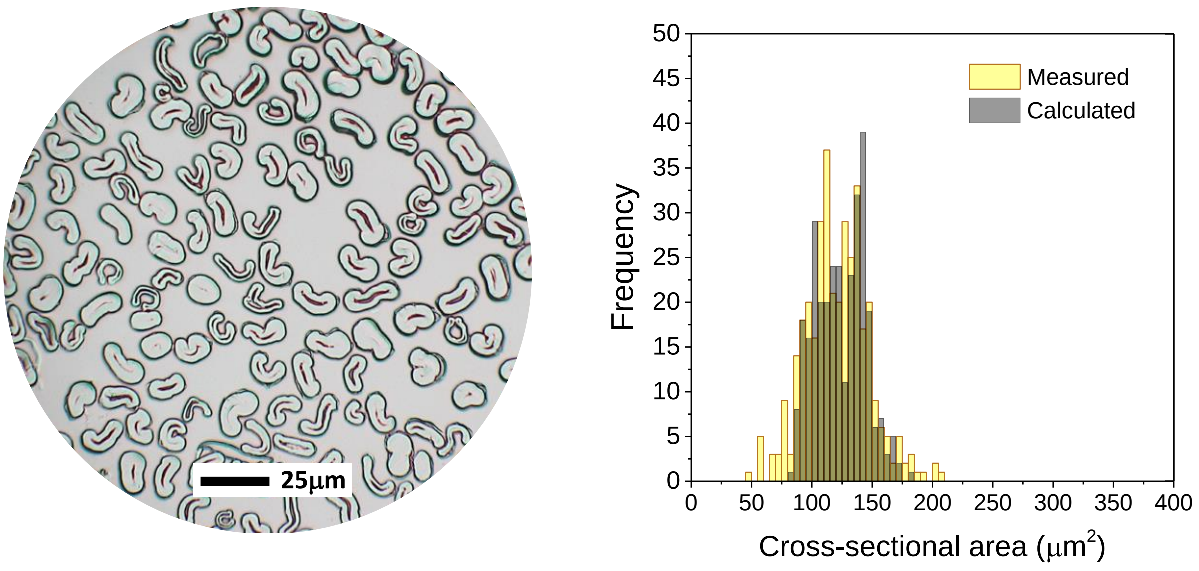

Figure S2. Optical miscroscopic image of the cross-sections and distributions of the cross-sectional areas measured by the image analysis and calculated from linear density for control cotton fiber.

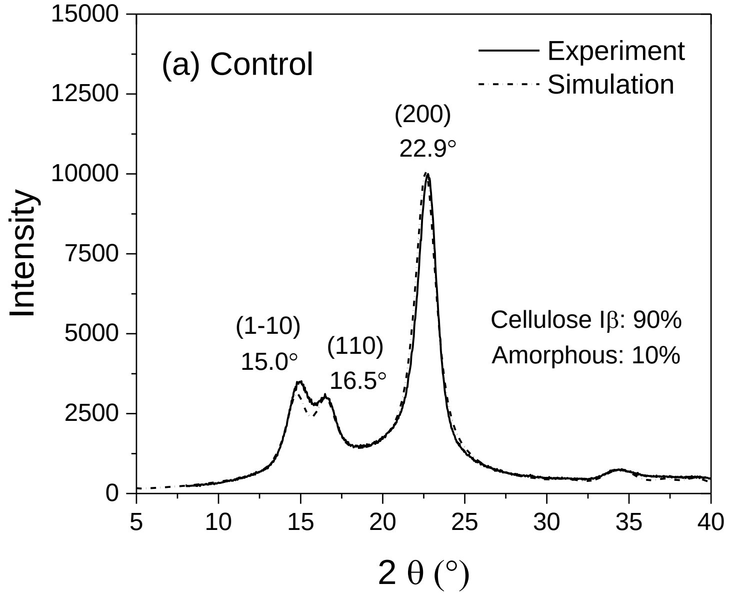

Figure S3. X-ray diffraction pattern of control cotton fiber plotted with the simulated pattern.

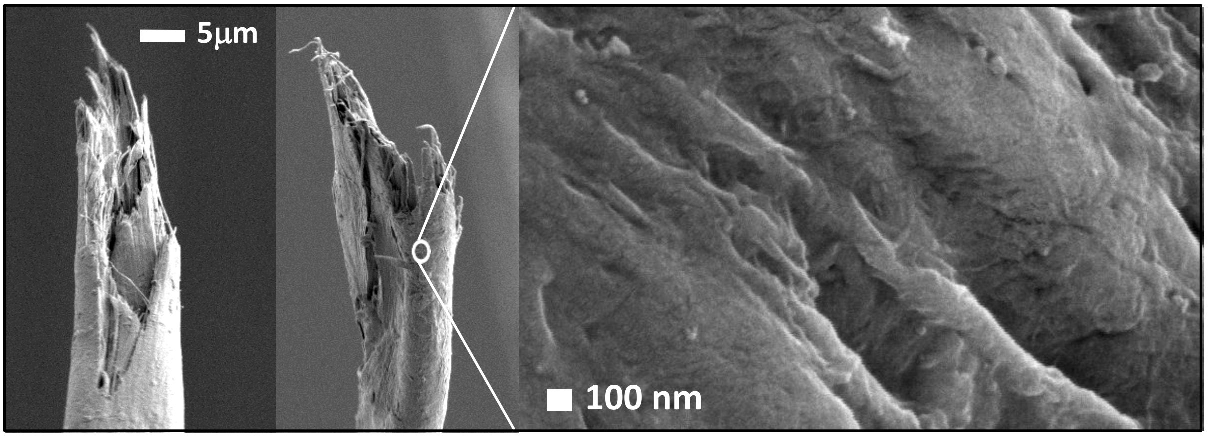

Figure S4. SEM images of the fractured control cotton fiber by tensile tests and a field emission SEM image of the surface for control cotton fiber taken at high magnification (50,000X).

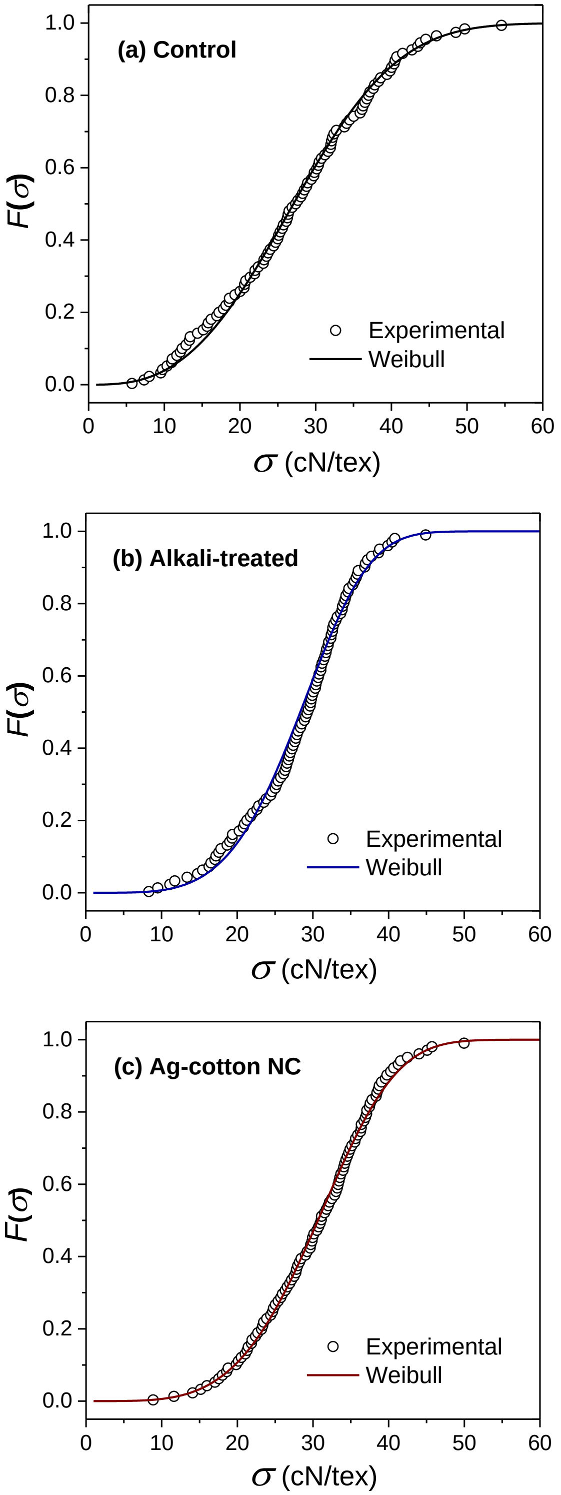

Figure S5. Empirical cumulative distributions of tenacity for control cotton fiber, alkali-treated cotton fiber, and Ag-cotton NC fiber plotted with two-parameter Weibull fits.
